# Primary Skin Fibroblasts as a Model of Parkinson's Disease

**DOI:** 10.1007/s12035-012-8245-1

**Published:** 2012-02-19

**Authors:** Georg Auburger, Michael Klinkenberg, Jessica Drost, Katrin Marcus, Blas Morales-Gordo, Wolfram S. Kunz, Ulrich Brandt, Vania Broccoli, Heinz Reichmann, Suzana Gispert, Marina Jendrach

**Affiliations:** 1 Experimental Neurology, Department of Neurology, Goethe University Hospital, Theodor Stern Kai 7, 60590 Frankfurt am Main, Germany; 2 Medical Proteome Center, Ruhr-University, Bochum, Germany; 3 Department of Neurology, University Hospital San Cecilio, Granada, Spain; 4 Department of Epileptology, University Bonn, Bonn, Germany; 5 Molecular Bioenergetics, Center of Biological Chemistry, Goethe University Hospital, Frankfurt am Main, Germany; 6 San Raffaele Scientific Institute, Milano, Italy; 7 Department of Neurology, University Clinic Carl Gustav Carus, Dresden, Germany

**Keywords:** Skin fibroblast, Parkinson's disease, PARK6, PARK2, PARK7, iPS

## Abstract

Parkinson's disease is the second most frequent neurodegenerative disorder. While most cases occur sporadic mutations in a growing number of genes including Parkin (PARK2) and PINK1 (PARK6) have been associated with the disease. Different animal models and cell models like patient skin fibroblasts and recombinant cell lines can be used as model systems for Parkinson's disease. Skin fibroblasts present a system with defined mutations and the cumulative cellular damage of the patients. PINK1 and Parkin genes show relevant expression levels in human fibroblasts and since both genes participate in stress response pathways, we believe fibroblasts advantageous in order to assess, e.g. the effect of stressors. Furthermore, since a bioenergetic deficit underlies early stage Parkinson's disease, while atrophy underlies later stages, the use of primary cells seems preferable over the use of tumor cell lines. The new option to use fibroblast-derived induced pluripotent stem cells redifferentiated into dopaminergic neurons is an additional benefit. However, the use of fibroblast has also some drawbacks. We have investigated PARK6 fibroblasts and they mirror closely the respiratory alterations, the expression profiles, the mitochondrial dynamics pathology and the vulnerability to proteasomal stress that has been documented in other model systems. Fibroblasts from patients with PARK2, PARK6, idiopathic Parkinson's disease, Alzheimer's disease, and spinocerebellar ataxia type 2 demonstrated a distinct and unique mRNA expression pattern of key genes in neurodegeneration. Thus, primary skin fibroblasts are a useful Parkinson's disease model, able to serve as a complement to animal mutants, transformed cell lines and patient tissues.

## Introduction

In Parkinson's disease (PD) research, past beliefs about a quite exclusive affection of the dopaminergic nigrostriatal pathway have been gradually superseded by neuropathological data on sporadic PD patients; in particular, the recent documentation of disease progression from gastrointestinal neurons via the brainstem/olfactory bulb onto the higher cortical regions [1, 2]. At present, data from genetic PD variants clearly indicate an early bioenergetic pathology also in extraneuronal tissues. Thus, in the future, it may become possible to objectively diagnose PD on the basis of a blood sample, saliva or a buccal epithelial swab, or a skin biopsy. We have explored several material sources and found primary skin fibroblast cultures a recommendable approach.

## Advantages and Disadvantages of Skin Fibroblasts as an In Vitro Model of PD

The main advantages of using skin fibroblasts as an in vitro model of PD are their availability and robustness. Furthermore, skin fibroblasts represent a model of primary human cells, which comprise the chronological and biological aging of the patients according to their polygenic predisposition and environmental etiopathology.

Skin fibroblasts can be easily isolated from 2 mm punch skin biopsies, a procedure, which does not need stitches and has practically as few complications as a venous puncture. Still, it should be performed by a dermatologist and is not a routine measure in the management of PD patients, thus requiring written consent and ethics commission approval.

The ensuing cell culture is a mixture of primary fibroblasts and keratinocytes at the beginning of the culturing process and a pure culture of fibroblasts is only achieved in the third passage. However, the fibroblast population consists most probably of a mixture of mitotic and postmitotic fibroblast [3], thus contributing to a heterogeneous cell population even at early passages. Furthermore, cells may be contaminated with the frequent skin microorganism *Mycoplasma*, possibly causing deprivation of nutrients, reduced growth, inflammatory responses, and oxidative stress, which makes a periodic testing for *Mycoplasma* necessary. Cell propagation, storage of aliquots in liquid nitrogen, and transport are easy and comparable to standard cell lines, so fibroblasts from patients with sporadic PD or with defined mutations of PARK genes can be obtained from numerous labs and several repositories such as the Coriell Institute in New Jersey.

Since clonal selection and drift in culture are inherent features of fibroblasts, the matching of fibroblasts from a sufficient number of patients with their appropriate controls of similar age and sex is always an inevitable difficulty. A possible measure to adjust controls to the patient fibroblasts could be the correction of diverting genes. Gene correction has been successfully applied to alter genes in induced pluripotent stem (iPS) cells [4] and fibroblasts [5, 6]. However, the possibility of off-target mutations is quite high, and a time-consuming prescreening process is necessary to determine the genes needing alterations.

In view of the slow growth of primary cells from aged individuals, it needs weeks in culture to generate sufficient material for a number of biochemical tests. After some cultivation time, primary skin fibroblasts may be similar to mouse embryonic fibroblasts (MEFs) which either transform spontaneously or reach replicative senescence, thus altering the previously established phenotypes. Therefore, as with all primary cell models, a careful documentation of culture history, number of population doublings, and senescence markers such as senescence-activated β-galactosidase (SA-β-galactosidase) staining are indispensable quality controls. Furthermore, control cells and patients fibroblast should have a similar amount of population doublings when comparing biochemical or genetic parameters. On the other hand, immortalization of fibroblasts can be regarded as an advantage since immortalized cells proliferate faster than primary cells, thus allowing a much higher cell yield, and characteristics induced by in vitro aging can be disregarded. A study by Sprenger et al. [7] comparing primary and immortalized fibroblasts shows that both cell types are quite similar in the early passages regarding “major cell lineage-specific characteristics” but expression changes of genes and proteins involved in transcription, cell cycle, receptor tyrosine kinase signaling cascade, and in the regulation of the cytoskeleton have been reported [7–11], indicating that the use of immortalized fibroblast for studies involving these pathways must be carefully controlled, e.g., by including primary fibroblasts.

The advantages and disadvantages of primary skin fibroblasts as an extraneural disease model are well established from previous research on Alzheimer's disease (AD), amyotrophic lateral sclerosis, Lesh–Nyhan syndrome, lysosomal and mitochondrial disorders, and aging and are summarized below based on previous reviews [12–15].

**Table:** 

**Arguments pro primary skin fibroblasts as a disease model**
•Easy availability from patients and matched controls, academic labs, cell repositories
•Robustness in culture, storage, and transport
•Mirror the polygenic risk factors of specific patients
•Reflect cumulative cell damage at the age of the patient
•Express most of the PARK genes at relevant levels
•Make dynamic cell contacts, similar to neurons and in contrast to most patient blood cells
•Can be reprogrammed to iPS cells and redifferentiated, e.g., to dopaminergic neurons as a human neuronal in vitro model of specific Parkinson variants
•As primary cells, they do not display maximal glycolysis (Warburg effect) and the independence from trophic signals which are typical of tumor cell lines
•Due to the homogenous cell differentiation, the signal-to-noise ratio is for many analyses better than in complex tissues such as brain
•Fibroblasts are quite amenable to genetic manipulation via electroporation or lentiviral constructs
•Human fibroblasts can be easily compared with mouse mutant embryonal fibroblasts
**Argumente contra primary skin fibroblasts as a disease model**
•Pure fibroblast culture only after passage 3, possible mixture of proliferating and postmitotic cells
•Population doubling time of patient and control fibroblasts must be closely monitored
•Growth especially in older populations is slow
•Suboptimal matching of patient cells with control cells, variances of seeding density, cell confluence and of substrate availability can generate irreproducible results
•Contaminations with *Mycoplasma* are frequent and may lead to artificial phenotypes
•Cells in culture have maximal trophic support, while neurons in vivo have to compete for it
•Fibroblasts are quite resistant against most stressors
•Their gene expression profile and their signaling differ strongly from neurons, e.g., the PD-associated gene alpha-synuclein is barely expressed; the vesicle/receptor/ion channel control, which is highly sophisticated in neurons is rather rudimentary in fibroblasts

## Identification of Potential Biomarkers for Diagnostics in Skin Fibroblasts

While patient skin fibroblasts can be obtained repeatedly with ease and thus might be used even to monitor disease progression, it still remains unclear to what extent they will be helpful to identify biomarkers for the diagnosis of predisposition and manifestation of PD individuals at risk (state and trait markers). The identification of objective molecular biomarkers for PD has been attempted in blood, serum, plasma, urine, and cerebrospinal fluid [16, 17]. To date, diagnosis of PD in hospital routine is still made subjectively on the basis of the clinical neurological examination and the response to specific drugs such as levodopa. In vivo objective diagnosis is so far limited to imaging investigations such as DATscan, which are not completely specific for PD and become pathological only around the time of clinical onset. The validation of diagnosis still depends on postmortem brain histology. In the context of skin fibroblasts, it is noteworthy that efforts to improve diagnostics of PD through chest skin biopsies yielded promising preliminary results, detecting alpha-synuclein containing Lewy neurites in a subset of cases, thus supporting the concept of PD as a systemic disease [18–22].

We have investigated the expression profile of primary skin fibroblasts with PINK1 (PARK6) and Parkin (PARK2) mutations at the global transcriptome and proteome level before generating and characterizing the appropriate mouse mutants [23–25] and were surprised to find only few strong transcript changes, but interestingly, the mRNAs of several other PARK genes such as alpha-synuclein and Parkin were dysregulated [23, 26]. However, due to the low expression of alpha-synuclein in fibroblasts, these data could not be analyzed at the protein level.

In an expansion of the fibroblast transcriptome analysis, the mRNA expression of 24 genes with key roles in neurodegeneration, especially in familial and sporadic PD, was analyzed in different fibroblast cultures. We compared fibroblasts of PARK2, PARK6, and of idiopathic Parkinson's disease (IPD) patients with fibroblasts from patients with familial and sporadic AD and spinocerebellar ataxia type 2 (SCA2). For normalization, age-matched control fibroblasts were used. Each fibroblast population demonstrated a unique expression profile where only a few common transcript changes in the different fibroblasts were observed (Table 1). Interestingly, expression patterns of PD fibroblasts were more similar to each other than to fibroblasts of AD and SCA2 patients. Furthermore, a distinct separation can be made between the IPD patient fibroblasts and the cultures of patients with familial PD. These data underline evidently the specificity of human fibroblasts as disease models.

**Table 1:** Distinct expression profiles of patient fibroblasts

Gene	Expression assay	PARK2	PARK6	IPD	AD	SCA2
Monogenic recessive Parkinsonism genes						
*PARKIN*	Hs01038318_m1	0.41 ± 0.10**	–^b,^ **	0.39 ± 0.08*	–	–
*PINK1*	Hs00260868_m1	1.33 ± 0.09**	–	–	–	–
*ATP13A2*	Hs00223032_m1	–	1.33 ± 0.07*	1.80 ± 0.02***	–	–
*PLA2G6*	Hs00185926_m1	–	0.54 ± 0.04**	0.39 ± 0.06**	–	–
*FBXO7*	Hs00201825_m1	–	0.79 ± 0.03*	0.75 ± 0.03**	0.82 ± 0.02*	1.23 ± 0.06*
*DJ-1*	Hs00697109_m1	–	–	–	–	–
Monogenic dominant Parkinsonism genes						
*SNCA*	Hs00240906_m1	n.d.	–*^, a^	–^a^	–^a^	n.d.
*LRRK2*	Hs00411197_m1	0.66 ± 0.08*	0.25 ± 0.03***	–	–	2.10 ± 0.34*
Other monogenic Parkinsonism genes						
*EIF4G1*	Hs00191933_m1	–	–	1.66 ± 0.08*	1.73 ± 0.15**	–
*OMI/HTRA2*	Hs00234883_m1	–	–	1.32 ± 0.03*	–	–
*VPS35*	Hs00372497_m1	–	–	–	–	–
*UCHL1*	Hs00188233_m1	–	–	–	–	–
*GIGYF2*	Hs01084510_m1	–	–	–	–	–
GWAS candidate Parkinsonism genes^c^						
*GAK*	Hs01049227_m1	–	–	1.45 ± 0.15*	–	–
*SYT11*	Hs01064643_m1	–	–	0.53 ± 0.04*	–	–
*BST1*	Hs01070189_m1	–	–	–	–	–
*HIP1R*	Hs00391321_m1	–	–	–	–	–
*STK39*	Hs01085346_m1	–	–	–	–	–
Other neurodegenerative disease genes						
*MAPT*	Hs00902194_m1	–	–	0.16 ± 0.04*	–	–
*BACE1*	Hs01121199_m1	–	0.87 ± 0.04*	1.19 ± 0.06*	1.39 ± 0.15*	–
*GBA*	Hs00164683_m1	–	–	–	–	–
*ATXN2*	Hs00268077_m1	–	–	–	–	–
*ATXN3*	Hs01026447_m1	–	–	–	–	–
*TARDBP*	Hs00606522_m1	–	–	–	–	–

The mRNA expression of the indicated genes with key roles in neurodegeneration, especially in PD, was analyzed in fibroblasts of PARK2, PARK6, and IPD patients and compared to fibroblasts of AD and SCA2 patients and normalized to age-matched controls. A unique expression pattern for each fibroblasts culture is visible, whereas the PD fibroblasts demonstrate clearly a different expression pattern in comparison to the AD and SCA2 fibroblasts. Analysis of mRNA levels between control and disease fibroblast cultures was performed by qPCR using TaqMan gene expression assays (Applied Biosystems, Darmstadt, Germany). Statistics were carried out by unpaired *t* test between fold changes of controls (*n* = 4) and the respective disease cultures

*PARK2* familial Parkinson's disease—V56E/C212Y-PARKIN (Hoenicka et al. [75]), *n* = 3; *PARK6* familial Parkinson's disease—G309D-PINK1 (Hoepken et al. 2007 [47]), *n* = 3; *IPD* idiopathic Parkinson's disease, *n* = 4; *AD* Alzheimer's disease (familial, *n* = 2; sporadic, *n* = 2); *SCA2* spinocerebellar ataxia type 2, *n* = 4; *GWAS* genome-wide association study (ACMSD, HLA-DRB, and LAMP3 were not detectable in fibroblasts); *n.d.* not determined

*P ≤ 0.05, **P ≤ 0.01, ***P ≤ 0.001

^a^Hoepken et al. [23]

^b^Klinkenberg et al. [26]

^c^Genome-wide association study (ACMSD, HLA-DRB, and LAMP3 were not detectable in fibroblasts)

In correlation with the transcript data described above, almost no consistent proteome changes in 2D-DIGE gels under culture conditions with maximal trophic support were found (unpublished data). In contrast, our data show very clear and consistent expression anomalies and mitochondrial pathology under distinct deprivation conditions, probably due to the fact that the function of PINK1 and Parkin is part of a quality control pathway that becomes relevant only under stress conditions [27–29].

Consequently, different groups have explored analyses in media with only 5% fetal calf serum, with low glucose [30], with galactose as carbon source [31], with rapamycin [32], in the presence of proteasome inhibitors [26, 33], after administration of complex I inhibitors or mitochondrial uncouplers [34, 35], or even in time course experiments after complete serum withdrawal [29]. The relevant stressors remain to be elucidated, underlining the necessity to define culture conditions that reliably mirror PD pathogenesis and are thus relevant for biomarkers. Interestingly, differing resistance to the respiratory chain complex I inhibitor rotenone characterizes skin fibroblast lines from different animal species, while variations in mitochondrial membrane potential are large in fibroblasts even within the same species. These mitochondrial differences correlate well with age within species and with life expectancy across species and importantly depend on nuclear factors [36–38]. Overall, nuclear and mitochondrial, genetic, and environmental factors contribute to the risk of PD. Therefore, a panel with both sensitive and specific state and trait diagnostic biomarkers will have to be developed from in vitro and in vivo approaches, on the basis of initial attempts in AD [39]. Also, it remains to be determined which biomarkers reflect progression and treatment response.

## Mechanistic Insights into PD Pathogenesis from Skin Fibroblasts

Observations that skin fibroblasts from sporadic PD patients show reduced respiratory complex I activity, pyruvate utilization, ATP generation, mitochondrial membrane potential, and increased lipid peroxidation similar to affected brain tissue were already made more than 20 years ago and were among the early evidence that the bioenergetic deficit typical of PD is not restricted to degenerating dopaminergic midbrain neurons, but an early systemic feature [40–44]. We have used skin fibroblasts successfully to rescue this bioenergetic deficit by pharmacological administration of CoQ(10) in approximately half of the cultures studied [45].

PARK6 patient skin fibroblasts represent a useful disease model as judged by their consistent respiratory deficit, altered mitochondrial morphology, increased oxidative stress, apoptosis vulnerability, and dysregulated expression of other PARK genes [23, 26, 30, 33, 46–50]. The analysis of PARK6 fibroblasts also contributed to the insight that PINK1 loss-of-function can be rescued by Parkin [30, 51], placing both proteins within a common pathway where stress-stabilized PINK1 acts upstream and regulates the stress-triggered mitochondrial translocation and degradation of Parkin.

In PARK2 patient skin fibroblasts with Parkin loss-of-function mutations, again a bioenergetic deficit with altered expression of nuclear-encoded mitochondrial proteins was documented. In addition, an enhanced mitochondrial vulnerability to DNA damage together with enhanced levels and activity of the DNA repair protein p53, as well as changes in the MAP kinase pathway and microtubule polymerization were observed [52–56].

In Parkin research, investigators only rarely resort to the transformed murine fibroblast cell line NIH3T3 with a specific knockdown as disease model [57, 58]; MEFs are unsuitable as model since they do not express Parkin. Due to the rarity of patients, the use of MEFs has become much more widespread in PARK7 research, documenting the DJ-1 loss-of-function to result in vulnerability to oxidative stress and in a loss of the transcription factor Nrf2. The data also indicated a cytoprotective role for the binding of DJ-1 to the apoptosis signal-regulating kinase 1 and an essential role of DJ-1 as oncogene on the upregulation of c-Myc [59–62]. Thus, at least for the autosomal recessive PD variants, the use of patient skin fibroblasts and of mouse mutant embryonal fibroblasts has quite faithfully modeled the known disease features and generated substantial mechanistic insights.

## The Use of Skin Fibroblasts for Transplantation and Reprogramming

Skin fibroblasts have been the basic tool for numerous PD treatment research efforts, which tested the benefit of retroviruses in mediating selective gene transfer and which assessed the benefit of reimplantation of genetically modified cells into brains with Parkinsonian neurodegeneration. Dopamine neurotransmission enzymes such as TH, GTPCHI, AADC, and VMAT2 and trophic factors such as GDNF and BDNF were successfully engineered into fibroblasts [63, 64]. Transplantation of such cells into neurotoxin rodent models of PD results in promising beneficial effects on the biochemical and behavior profile [65–68].

Great promise is now derived from recent achievements in the field where mouse and PD patient fibroblasts were reprogrammed into adult iPS cells [69, 70], which can be redifferentiated into neuronal cells with dopaminergic characteristics [71, 72], and can be integrated successfully into the fetal brain with a beneficial effect on symptoms of the neurotoxic rat PD model [73]. Efforts are now underway to rescue the autosomal recessive PD variants in the skin fibroblast model and use the derived dopaminergic neurons as material for the identification of biomarkers and mechanistic insights. Since iPS-derived cells may be genomically unstable [74], it remains unclear to what extent they might be useful for future transplantation trials in patients. Furthermore, candidate disease phenotypes observed in iPS-derived cells will have to be validated in tissues or primary cells from patients.

In conclusion, we regard skin fibroblasts as a useful and promising complement to the better established analyses of patient or mouse mutant tissues and to the widespread use of transfected tumor cell lines. Therefore, we would like to encourage academically interested clinicians to obtain skin biopsies from interesting PD patients and their relatives to make them available internationally for academic research.

## Abbreviations

AD: Alzheimer's disease
iPS: Induced pluripotent stem cells
IPD: Idiopathic Parkinson's disease
MEF: Mouse embryonic fibroblasts
PINK1: PTEN-induced putative kinase 1
PD: Parkinson's disease
SA-beta-galactosidase: Senescence-activated beta-galactosidase
SCA2: Spinocerebellar ataxia type 2
